# Molecular Characterization of H5N1 Clade 2.3.4.4b Virus in Vaccinated Layer Chickens

**DOI:** 10.3390/v18060589

**Published:** 2026-05-22

**Authors:** Ahmed H. Salaheldin, Mustafa Ozan Atasoy, Juliane Lang, Ann Kathrin Ahrens, Anne Pohlmann, Mohammed A. Rohaim, Hatem S. Abd El-Hamid, Elsayed M. Abdelwhab

**Affiliations:** 1 Department of Poultry and Fish Diseases, Faculty of Veterinary Medicine, Alexandria University, Edfina 22758, Egypt; 2 Department of Virology, Faculty of Veterinary Medicine, Cumhuriyet University, Sivas 58040, Türkiye; mozan@cumhuriyet.edu.tr; 3 Institute of Molecular Virology and Cell Biology, Federal Research Institute for Animal Health, Friedrich-Loeffler-Institut, 17493 Greifswald-Insel Riems, Germany; 1000000531.gast@fli.de; 4 Institute of Diagnostic Virology, Federal Research Institute for Animal Health, Friedrich-Loeffler-Institut, 17493 Greifswald-Insel Riems, Germany; annkathrin.ahrens@fli.de (A.K.A.); anne.pohlmann@fli.de (A.P.); 5 Department of Virology, Faculty of Veterinary Medicine, Cairo University, Giza 12211, Egypt; mohammed_abdelmohsen@cu.edu.eg; 6 Department of Poultry and Fish Diseases, Faculty of Veterinary Medicine, Damanhour University, Damanhour 22511, Egypt; hatem.s.abdel-hamid@vetmed.dmu.edu.eg

**Keywords:** vaccine breakthrough, H5N1 clade 2.3.4.4b, layers, antigenic drift, antibody titers, Egypt

## Abstract

The global emergence of the avian influenza virus (AIV) H5N1 clade 2.3.4.4b since 2016 has caused substantial losses in wild bird and poultry populations, along with heightened risks of transmission to humans and other mammals. Vaccination of poultry has been a key strategy to curb the virus’s spread and mitigate its socioeconomic impact. This report describes an outbreak of high pathogenicity avian influenza virus (HPAIV) H5N1 clade 2.3.4.4b in a flock of 15,000 brown layer chickens (170 days old), all of which had received a four-dose vaccination regimen with H5N1/H5N8 commercial vaccines at 17, 50, 100, and 125 days of age. Despite this vaccination history, H5N1 infection was confirmed approximately seven weeks post-vaccination. H5N1 infection was confirmed by RT-qPCR, virus isolation, and full genome sequencing covering all eight gene segments, followed by phylogenetic and molecular analyses. Clinical signs included reduced feed intake, decreased egg production, and a cumulative mortality rate of 35% over 52 days. Hemagglutination inhibition (HI) testing with various H5 antigens revealed inconsistent antibody titers (geometric mean: 4.0 to 9.1 log2). Genetic analysis of the full-length HA and NA gene sequences further revealed strong similarity to contemporaneous H5N1 clade 2.3.4.4b strains circulating in Egypt, with multiple mutations in the HA head domain, particularly near immunogenic epitopes and receptor binding sites. These findings highlight the limitations of current vaccination strategies under conditions of antigenic mismatch and complex immunization schedules, emphasizing the need for improved vaccine matching and continuous molecular surveillance. To improve outbreak management in poultry, enhanced vaccination protocols, stringent biosecurity measures, and rigorous monitoring practices are critical.

## 1. Introduction

Layer chickens represent a critical component of global food systems, providing a reliable and nutritious source of eggs that supply high-quality protein, essential vitamins, and minerals for human diets [1]. Egg production also supports millions of livelihoods across the poultry production chain and contributes significantly to global agricultural economies. Global egg production increased from approximately 15 million tons in 1961 to 93 million tons in 2020, highlighting the growing importance of layer chickens in meeting rising global demand for animal protein, particularly in developing countries such as Egypt [2,3]. However, poultry production remains highly vulnerable to infectious diseases, among which avian influenza viruses (AIV) represent one of the most significant threats worldwide [4]. AIV are classified according to their surface glycoproteins hemagglutinin (HA) and neuraminidase (NA), comprising 17 HA and 9 NA subtypes. Although wild aquatic birds represent the natural reservoir where infections are typically asymptomatic, spillover into domestic poultry can result in low pathogenic (LP) infections or highly pathogenic avian influenza (HPAI) outbreaks associated with severe economic losses [5]. Since its emergence in 1996/1997, the HPAI H5 subtype belonging to the Goose/Guangdong lineage has caused extensive outbreaks and diversified into multiple phylogenetic clades [6]. More recently, the emergence and global spread of H5Nx viruses of clade 2.3.4.4b since 2016 has aggravated the epidemiological situation, with viruses disseminating from Asia into Europe, Africa, and North America and demonstrating the capacity to infect a range of mammalian hosts including humans [7,8,9,10,11,12]. Thus, effective control of avian influenza in poultry remains essential to limit economic losses and reduce the risk of zoonotic transmission [13].

Egypt is considered an important epidemiological hotspot for avian influenza because of its dense poultry population and its position within established migratory bird corridors, which may facilitate long-distance ecological connectivity between Eurasia and Africa [14]. Several zoonotic AIV subtypes have been detected in Egyptian poultry, including H5N1 clade 2.2.1 (2005–2019), H9N2 (since 2013), H5N8 clade 2.3.4.4b (since 2016/2017), and H5N1 clade 2.3.4.4b (since 2020) [15,16,17]. The introduction of H5N8 viruses through migratory birds led to their rapid dissemination in poultry farms, replacing the previously endemic H5N1 clade 2.2.1 lineage, while H5N1 clade 2.3.4.4b has recently become dominant over H5N8 in Egypt. The country’s position along migratory flyways connecting Eurasia and Africa therefore continues to facilitate the introduction and spread of AIV strains [18,19]. Egypt also reports the highest global number of human H5N1 infections, together with sporadic human cases of H9N2 infection [20,21].

To mitigate the socioeconomic impact of avian influenza outbreaks, Egypt implemented extensive H5 vaccination programmes in poultry production systems. Following the first detection of HPAI H5N1 in February 2006, a nationwide vaccination programme was initiated in May 2006, with projected costs approaching 24 million USD within three years [22,23,24,25,26,27]. Early investigations demonstrated that viruses circulating between 2006 and 2009 belonged to the Eurasian lineage H5N1 clade 2.2.1 and subsequently evolved through antigenic drift [28]. However, the first vaccine strains deployed, including Gs/GD (H5N1) and CK/Mexico/232/94 (H5N2), exhibited limited cross-reactivity with circulating field viruses, prompting ongoing debate regarding vaccination strategies. While some studies suggested that vaccination may facilitate the emergence of antigenically drifted variants despite providing clinical protection, others reported no association between vaccination and viral evolution and emphasised the continued importance of vaccination in reducing disease severity and production losses [28,29,30,31,32]. Despite extensive vaccination efforts, H5N1 and H9N2 viruses continue to be detected in vaccinated flocks. Therefore, in the present study we aimed to characterise cases associated with a suspected vaccine-escape H5N1 virus detected in layer chickens that had received four different H5 vaccinations, integrating clinical observations, mortality data, molecular analyses, and in silico characterisation.

## 2. Materials and Methods

### 2.1. Background of AIV Outbreak and Sample Collection

In mid-2024, an outbreak was detected in the grower unit located in Alexandria belonging to a commercial poultry production company. The affected holding comprised 15,000 brown layer chickens aged 170 days, housed in four rows of cages. According to farm records, the flock had been vaccinated against avian influenza under a multi-dose program, H5N1 Re5 (clade 2.3.4) at 17 days of age, H5N1 Re8 (clade 2.3.4.4g) at 50 days of age, H5N6 Re13 (clade 2.3.4.4h) and H5N8 Re14 (clade 2.3.4.4b) at 100 days of age, and H5N8 GWT (clade 2.3.4.4b) at 125 days of age. H5 avian influenza infection was laboratory-confirmed on 1 September 2024. Subsequent real-time RT-PCR testing performed on 8 September, and 14 October 2024 demonstrated ongoing viral circulation. Following the onset of infection, the flock exhibited a marked decline in feed intake and production performance, accompanied by increased mortality. Daily mortality initially involved three birds, subsequently escalating and reaching a peak of approximately 200 birds in one of the four rows within the affected unit. Swab and serum samples were collected from the flock by a local veterinary practitioner and immediately submitted to the laboratory. Swabs were collected from the cloaca and oropharynx of 25 birds that exhibited clinical signs or found dead. These swabs were placed in a virus transport medium and sent to the Faculty of Veterinary Medicine at Damanhur University, Egypt, for routine diagnosis. Blood samples were collected from 30 chickens on different intervals after vaccination.

### 2.2. Virus Detection and Isolation

Field materials were centrifuged at 3000× *g* for 5 min to remove cellular debris and sterile filtered through a 0.45 µm PVDF syringe filter prior to inoculation. An aliquot of 250 µL of the clarified supernatant was collected from each sample for downstream analyses, and the remaining material was stored at −80 °C for further characterisation. Total RNA was extracted using TRIzol™ LS Reagent (Thermo Fisher Scientific, Waltham, MA, USA) in accordance with the manufacturer’s instructions. The extracted RNA underwent preliminary quality assessment using a NanoDrop spectrophotometer (Thermo Fisher Scientific, Waltham, MA, USA), with A260/280 ratios evaluated to confirm adequate purity and the absence of residual phenol. Detection of influenza A virus was performed using a one-step real-time RT-qPCR assay targeting the conserved matrix (M) gene, using primers and probes described previously [33]. Positive samples were further analysed for HA and NA subtype identification using subtype-specific RT-qPCR-based assays described by Hoffmann et al. [34], performed on an Applied Biosystems 7500 Real-Time PCR System (Thermo Fisher Scientific, Waltham, MA, USA).

AIV-positive field materials were processed for virus isolation as described in the WOAH Terrestrial Manual guidelines [35]. Briefly, samples were inoculated into 10–12-day-old specific pathogen free embryonated chicken eggs through the allantoic cavity [36], incubated at 37 °C and 55–60% humidity for 2–3 days, and monitored daily by candling. Allantoic fluids were assessed for the presence of virus using the hemagglutination assay, in accordance with the standards determined by the WOAH [37]. No experimental animal research was conducted in this study, and all procedures involving chickens were carried out in compliance with applicable standard guidelines.

### 2.3. MiniON Sequencing and Data Processing

Nucleotide sequences of all genes were amplified using Rapid multiplex MinION nanopore sequencing workflow for Influenza A viruses as previously published by King et al. [38]. Briefly, viral RNA was initially amplified using primers reported by Hoffmann et al. [39], via conventional PCR reaction using one-step RT-PCR Kit (SuperScript™ III One-Step RT-PCR System with Platinum™ Taq DNA Polymerase; Invitrogen, Waltham, MA, USA). Then, amplicons were purified through AMPure XP Magnetic Beads (Beckman Coulter, Fullerton, CA, USA) and quality-assured using Qubit. Purified amplicons were barcoded and pooled using The Rapid Barcoding Kit 24 V14 (SQK-RBK114.24, ONT, Oxford, UK), and implemented into FLO-MIN114 flow cell following the manufacturer’s instructions.

### 2.4. Sequence and Phylogenetic Analyses

Data obtained from whole-genome sequencing were evaluated using multiple sequence analyses (MSAs). For this purpose, sequence data were retrieved from the GISAID EpiFlu™ platform (https://platform.gisaid.org/, accessed on 11 March 2026) [40], from which a dataset comprising H5Nx influenza virus sequences reported from the African continent and countries neighbouring Egypt, namely Israel, Jordan, Saudi Arabia, Iraq, and Syria, during the preceding five-year period was constructed. Then, each gene segment was initially screened against the whole NCBI and GISAID databases using the BLAST tool (https://blast.ncbi.nlm.nih.gov, accessed on 11 March 2026) [41], and the most closely related sequences available from the beginning of 2023 to date were also included in the data. To enrich the dataset and improve phylogenetic resolution, representative H5N1 strains showing high sequence identity and broad geographic distribution across Asia, Middle East, and Europe were additionally included in the analysis, enabling a more comprehensive comparative and evolutionary assessment. Respective sequences were aligned using the MAFFT v7.490 algorithm [42] implemented in Geneious Prime^®^ 2026.0.1 [43], and domain- and motif-specific annotations were evaluated at both the nucleotide and deduced amino acid levels.

Phylogenetic trees were generated for each gene segment using the PhyML v3.3.20180621 algorithm [44], with branch support assessed by 1000 bootstrap replicates. The optimal nucleotide substitution model for each respective segment was selected based on the Bayesian Information Criterion (BIC) following model testing performed in MEGA11 v11.0.13 [45]. For the FUBAR [46] and mutation distribution frequency analyses, all available Egyptian strains together with the AHS-M strain were included in the dataset. Default Bayesian settings were applied in FUBAR to model variation in substitution rates across sites, and mutation patterns were analysed using RStudio (v. 2024.09.0+375). Phylogenetic analysis was employed to investigate the evolutionary relationships of the detected strain, as it remains the standard approach for influenza virus classification and lineage tracking. While alternative methods such as principal component analysis (PCA) can provide useful insights into sequence variation, they are generally complementary rather than substitutive for phylogenetic inference. Future studies integrating both approaches may provide additional resolution regarding nucleotide-level variation and clustering patterns.

## 3. Results

### 3.1. Progress of H5 Outbreak

An H5 infection was confirmed on 1 September 2024, with subsequent RT-qPCR testing on 8 September and 14 October. Following confirmation of the H5 infection, feed intake and egg production declined, and mortality rates rose, starting at 3 birds and peaking at 200 birds in one of four rows. Mortality persisted for 52 days, affecting 35% of the flock. Whole-flock culling was not implemented in this case, potentially reflecting delayed diagnosis and field-level management considerations.

**Figure 1:**
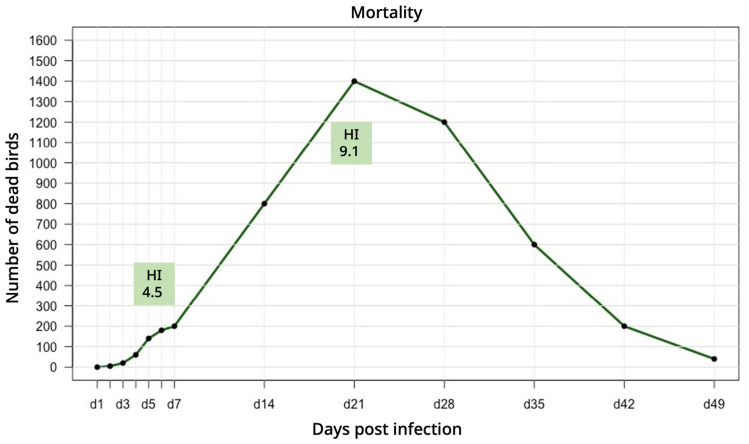
Daily mortality following H5 infection and corresponding haemagglutination inhibition (HI) antibody responses over time. Mortality increased progressively, peaking at day 21 post-infection, followed by a gradual decline until day 49. HI titres (geometric mean [GM] range: 4.5–9.1) against Re8 and Re13 antigens fluctuated over the monitoring period, with higher titres observed around the peak mortality phase. Mortality is shown as the number of deaths per day, and HI titres are presented at selected time points.

Hemagglutination inhibition (HI) titres measured against Re8 and Re13 antigens demonstrated fluctuating antibody responses over time, with geometric mean (GM) titres ranging from approximately 4.5 to 9.1 log_2_ (Figure 1). Notably, peak HI titres coincided with peak mortality, suggesting a delayed and likely infection-driven antibody response rather than fully protective pre-existing immunity. Additionally, HI testing against the Re14 antigen demonstrated moderate titres, with a calculated GMT of 3.6 log_2_, suggesting a relatively modest antibody response despite the high genetic relatedness to the AHS-M strain. This suggests that, under field conditions, the humoral response against the most closely matched vaccine antigen was heterogeneous and potentially insufficient to confer consistent protection.

### 3.2. Virus Isolation

Blind virus propagation in embryonated fowl eggs resulted in the death of all embryos within 48 h post-inoculation (hpi). Allantoic fluid from inoculated eggs was subsequently used for RNA isolation, and RT-qPCR targeting M gene further confirmed the presence of AIV. The virus was designated A/Chicken/Egypt/AHS-M/2024(H5N1) (hereafter referred to as AHS-M).

### 3.3. Molecular Characterisation of Novel AIVs

Genomic sequencing of the novel H5 AIV strain AHS-M confirmed all eight gene segments, with the following lengths: PB2 2340 bp, PB1 917 bp, PA 1917 bp, HA 1755 bp, NP 1552 bp, NA 1446 bp, M 1013 bp, and NS 874 bp (GISAID accession ID EPI_ISL_20438513).

#### 3.3.1. Hemagglutinin Gene

**Figure 2:**
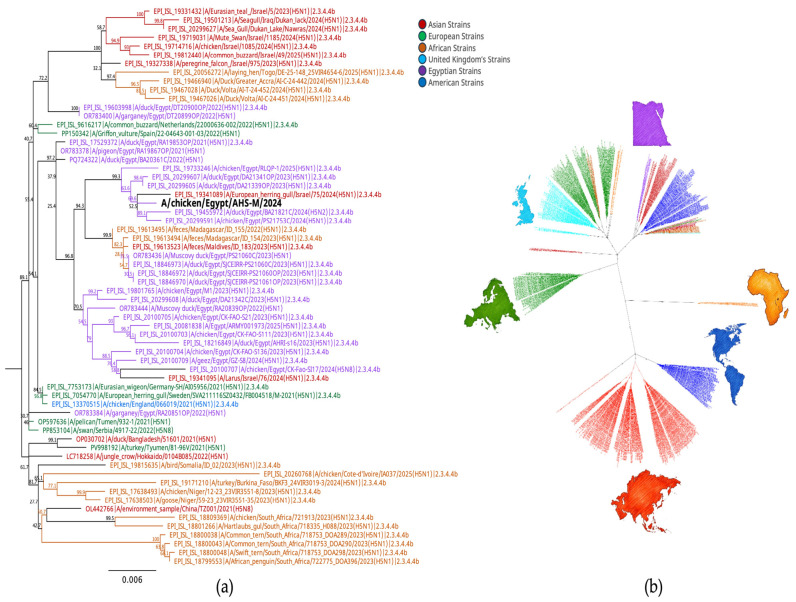
(**a**) Maximum-likelihood phylogenetic tree of complete hemagglutinin (HA) gene sequences inferred using PhyML under the TN93 nucleotide substitution model. The novel AHS-M strain is highlighted in bold black and clusters with previously reported Egyptian strains from chickens and ducks. Branch support was assessed with 1000 bootstrap replicates. (**b**) Unrooted phylogenetic tree based on sequence identity of AHS-M, illustrating global distribution and continental clustering of H5 sequences.

BLAST analysis of the HA gene segment identified strain AHS-M as a HPAIV of the H5N1 subtype classified within clade 2.3.4.4b. Phylogenetic analysis further showed that the AHS-M strain clustered closely with recently circulating H5N1 and H5N2 viruses detected in the Middle East and Egypt (Figure 2a,b). Distance matrix analysis further indicated that AHS-M shared the highest nucleotide identity (99.53%) with the HPAIV strain A/duck/Egypt/DA21343OP/2023 (EPI ID: EPI_ISL_20299607). In the phylogenetic tree, Egyptian strains segregated into two major groups with strong bootstrap support; one of these groups was further subdivided into two distinct subclusters that included AHS-M together with H5N1 and H5N2 viruses isolated from Egyptian ducks, as well as a single H5N1 strain (EPI ID: EPI_ISL_19341089) detected in a gull (*Larus argentatus*) in Israel in January 2024.

**Figure 3:**
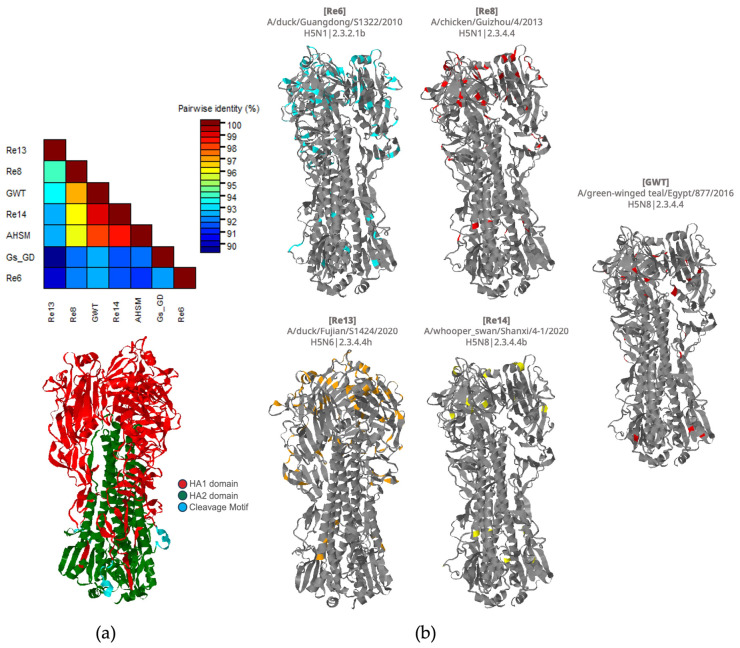
(**a**) Sequence demarcation analysis illustrating the pairwise amino acid identity (%) of the mature HA protein between AHS-M and representative vaccine strains (upper panel). The lower panel shows a schematic representation of the HA protein, highlighting the HA1 and HA2 domains and the cleavage motif using distinct colours. (**b**) Structural mapping of cumulative point mutations identified in the HA gene based on pairwise comparisons between the novel AHS-M variant and the respective vaccine strains. Amino acid substitutions unique to AHS-M were projected onto the corresponding vaccine strain structures, with different colours used to distinguish mutational differences.

The 567-amino acid HA sequence of AHS-M was annotated to identify functional domains and conserved motifs, enabling characterization of the structural features of the HA protein. The virus possessed a polybasic cleavage motif at the HA1–HA2 junction (PLRERRRKR↓G), a motif considered relatively uncommon within Egyptian strains and reported predominantly in H5N8 subtypes. Pairwise amino acid identity analysis revealed that the AHS-M isolate shared the highest similarity with the Re14 vaccine strain (98.41%), followed by Re8 (~96%), whereas the Re6 vaccine strain exhibited the lowest similarity (91.02%) (Figure 3a). Despite this high genetic similarity (98.41%), HI titres against Re14 remained moderate, with a GM of approximately 3.6 log_2_ and individual titres ranging from 3 to 5 log_2_. This suggests that genetic relatedness alone did not translate into consistently protective antibody responses under field conditions.

Three-dimensional modelling of the HA protein using the SWISS-MODEL server revealed several amino acid substitutions clustered within the globular head region of the HA1 subunit, particularly near immunogenic epitopes and receptor-binding sites. These included Lys189 located in the 190-helix region and Lys372 within the stalk domain. In addition, substitutions were identified within the signal peptide region, which has previously been reported to be enriched among circulating H5N1 viruses (Figure 3b).

**Figure 4:**
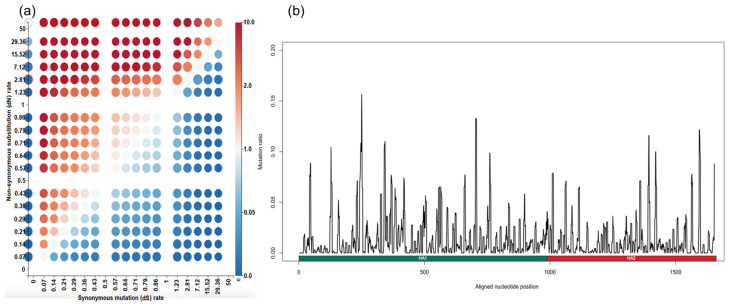
(**a**) Posterior distribution of synonymous (dS) and non-synonymous (dN) substitution rates across the complete HA gene of Egyptian influenza virus strains reported to date. The plot shows the posterior probability mass over a discretized grid of dS and dN values. Circle size is proportional to the posterior weight assigned to each rate combination, whereas colour represents the inferred selection intensity, expressed as ω = dN/dS. Blue denotes purifying selection (ω < 1), white indicates approximately neutral evolution (ω ≈ 1), and red indicates positive selection (ω > 1). (**b**) Distribution of mutation frequency along the mature HA protein of AHS-M compared with strains of Egyptian territory circulating over the last five years. Mutational events were predominantly concentrated within the HA1 subunit (green). The highest observed mutation ratio reached approximately 0.15 (i.e., 15% variability at a given nucleotide position among the aligned sequences).

Analysis of the complete HA gene of Egyptian strains revealed a heterogeneous distribution of synonymous (dS) and non-synonymous (dN) substitution rates across the discretized grid. Most of the posterior weight was concentrated in regions where dN exceeded dS (ω > 1), indicating that a substantial proportion of sites are evolving under positive selection. In contrast, regions where dN < dS (ω < 1) reflected purifying selection with lower posterior support. Only a limited number of rate combinations clustered around ω ≈ 1, suggesting that strictly neutral evolution plays a minor role. Overall, these results indicate that while purifying selection constrains parts of the HA gene, several sites show signatures of adaptive evolution (Figure 4a). Furthermore, HA sequences were further compared with other Egyptian strains including AHS-M reported so far to evaluate the cumulative mutation load. The mutation frequencies were evenly distributed between HA1 and HA2, with the profile showing fluctuations reaching up to 0.15 (approximately 15 mutations per 100 sequences) (Figure 4b).

#### 3.3.2. Neuraminidase Gene

**Figure 5:**
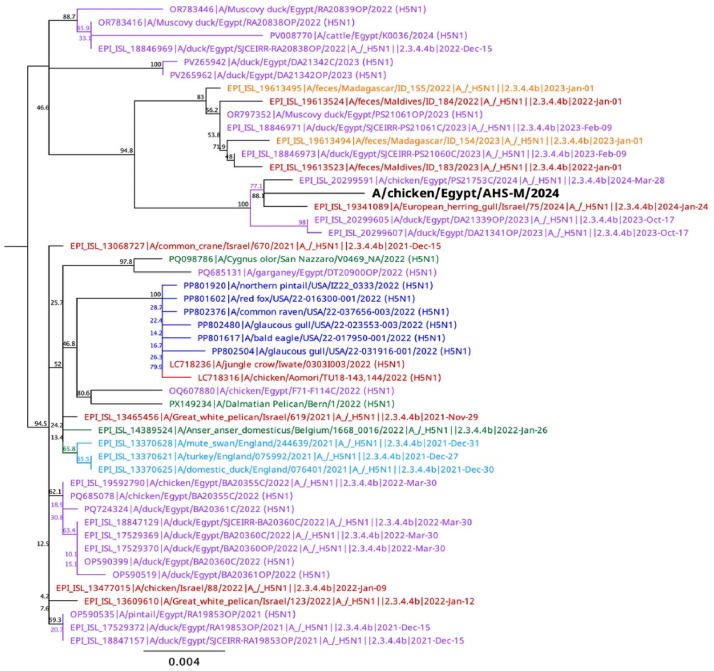
Maximum-likelihood phylogenetic tree of complete neuraminidase (NA) gene sequences constructed using PhyML under the HKY85 substitution model. Branch support was assessed by bootstrap analysis. Both strains cluster within clade 2.3.4.4b alongside recent Egyptian poultry isolates and a related Israeli wild-bird strain, with Egyptian viruses forming two distinct clusters.

The NA gene segment of strain AHS-M showed a close genetic relationship with contemporary regional H5N1 viruses based on BLAST analysis. Sequence comparison indicated that the NA gene shared the highest nucleotide identity with the clade 2.3.4.4b strain A/European_herring_gull/Israel/75/2024 (ISL_19341089), detected in a gull (*Larus argentatus*). Phylogenetic analysis supported this relationship, showing that AHS-M clustered within a subclade comprising Egyptian duck and chicken isolates collected during the 2023–2024 winter period, together with a single Israeli strain detected in a wild bird (Figure 5). Overall, Egyptian strains segregated into two major clusters with strong bootstrap support.

To further contextualize these findings, additional NA sequences from previously reported Egyptian strains were retrieved for comparative analysis. The analysis confirmed the absence of deletions in the NA stalk region of AHS-M, although such deletions have frequently been reported among Egyptian isolates. Comparative analysis of the 469-amino acid NA protein indicated a high degree of conservation, with only a small number of amino acid substitutions identified. These included Lys217Arg, Ile304Val, and Ile394Val. Among these, Ile304Val was commonly observed in Egyptian strains, whereas Ile394Val occurred less frequently. In contrast, residue 217 appeared less conserved, and the Lys217Arg substitution was rare, being detected in only one additional strain (A/Egy-CK-CV10OS-2023-N1; EPI3333776).

We further retrieved sequence data from other Egyptian strains to contextualise our findings. Analysis confirmed the absence of deletions within the NA stalk region, although such deletions have been frequently reported among Egyptian isolates. Comparative analysis of the NA protein sequences of the novel strains demonstrated a high degree of conservation, sharing 99.36% amino acid identity. Only three amino acid substitutions were identified, all of which were conservative, including Lys217Arg, Ile304Val, and Ile394Val. The Ile304Val substitution was commonly observed among Egyptian strains, whereas Ile394Val occurred less frequently. In contrast, residue 217 exhibited lower conservation, and the Lys217Arg substitution was rare, being detected in only one additional strain (A/CHICKEN/Egypt/A-Egy-CK-CV10OS-2023-N1/2023; EPI3333776).

## 4. Discussion

Since the emergence of HPAIV H5N1 (Gs/GD lineage) in Southeast China in the mid-1990s, descendant viruses have evolved and spread globally along migratory bird routes, resulting in a panzootic [47]. Clade 2.3.4.4b viruses are now enzootic in wild birds, facilitating recurrent outbreaks, and the emergence of novel reassortants, thereby causing substantial impacts on the poultry sector [48,49,50]. Recent reports of HPAI H5N1 in dairy cattle in the United States highlight potential risks of virus spread via contaminated products [51,52].

Vaccination remains a cornerstone of HPAIV control, reducing morbidity, mortality, and viral excretion [53]. In particular, advancements in reverse genetics have optimised influenza vaccine production by enabling the generation of recombinant viruses with predetermined protein composition, reducing reliance on labour-intensive reassortment workflows, and allowing more precise genetic matching to circulating strains, thereby helping to mitigate antigenic mismatch [54,55,56,57]. Furthermore, site-specific mutagenesis approaches have been developed to enhance vaccine safety while preserving protective efficacy and improving genetic stability [57,58,59]. However, its effectiveness can be compromised not only by antigenic mismatch, but also by factors such as improper administration, cold-chain failures, and insufficient biosecurity and quarantine measures [24,60,61]. In densely populated poultry regions like Egypt, these factors contribute to the persistence and evolution of antigenically drifted viruses within vaccinated flocks. Earlier HPAI subtypes belonging to clade 2.3.4, including H5N2, H5N5, and H5N8, were initially detected in poultry markets in China and subsequently spread across the Middle East and North Africa through migratory birds [8,62,63,64,65,66]. The continuous emergence of novel variants has necessitated constant monitoring and regular updating of vaccine seed strains [67]. In Egypt, several commercial vaccines derived from H5 subtype, including clade 2.3.4.4 viruses, are currently in use [68,69]. Within this context, our study aimed to investigate the molecular characteristics of recently circulating H5N1 viruses in Egyptian poultry and assess potential antigenic divergence from currently used vaccine strains. The observed outbreak dynamics should also be interpreted in light of field conditions. Delayed diagnosis, combined with ongoing production and variable implementation of control measures, may allow continued viral circulation within affected flocks. Such conditions can contribute to prolonged outbreaks and may increase the risk of transmission if biosecurity practices are insufficient.

Our phylogenetic analyses placed the novel AHS-M strain within clade 2.3.4.4b, clustering with recent Egyptian and regional isolates from both poultry and wild birds. This clustering is consistent with ongoing regional circulation of clade 2.3.4.4b viruses and may reflect both local virus persistence (e.g., in vaccinated poultry, backyard flocks, live bird markets, environmental reservoirs, and feral birds) and repeated introductions associated with wild bird movements along major migratory flyways linking Eurasia, the Middle East, and Africa. Previous studies have suggested that migratory birds contribute to the evolution, diversity, and dissemination of influenza viruses [70,71]. In line with this, Fang et al. reported that viral spread patterns can correspond to migratory flyways, with spatial and seasonal concordance observed across the United States between 2022 and 2025 [72]. In contrast, a PCA-based multivariate analysis of H5 avian influenza sequences revealed clear geographic clustering, indicating strong regional persistence with limited long-distance spread via migratory birds, and suggesting that conventional phylogenetic approaches may be insufficient to capture complex viral relationships [73,74]. Overall, the observed genetic similarity suggests ongoing regional circulation of clade 2.3.4.4b viruses rather than emergence of a distinct novel lineage. This interpretation is consistent with Egypt’s role as an ecological interface between wild reservoirs and commercial poultry populations [66,75,76,77,78,79]. Nevertheless, increased genomic surveillance is needed to better resolve the relative contributions of local persistence and repeated introductions.

Several amino acid substitutions identified in the HA protein have potential implications for antigenic drift and reduced vaccine effectiveness under field conditions. Notably, the Lys372Arg substitution occurs within residues 363–382, a region previously described as a broadly reactive epitope targeted by neutralising antibodies [80,81]. Additional polymorphisms were observed in antigenic site B and the 190-helix, both critical for host receptor engagement and immune recognition [82,83]. Mutations in these regions have been associated with reduced vaccine efficacy via antigenic drift [80]. Pairwise comparisons revealed that the HA sequence of the Re14 vaccine strain showed high amino acid similarity to AHS-M (98.41%), followed by Re8 (~96%). This divergence may have contributed to the fluctuating and generally low HI titres observed in the affected flock, despite multiple vaccinations. While functional confirmation through challenge studies is needed, these molecular signatures underscore the ongoing evolutionary dynamics of H5N1 in Egyptian poultry. The temporal dynamics observed in this study further support this interpretation. HI titres increased progressively and peaked at the same time as maximum mortality, indicating that the antibody response was likely infection-driven rather than solely vaccine-induced. This delayed immune response may have been insufficient to prevent disease progression, highlighting the limitations of pre-existing immunity in this flock despite repeated vaccination.

An additional factor that should be considered is the sequential use of multiple heterologous H5 vaccine strains (Re5, Re8, Re13, and Re14). While this approach is intended to broaden antigenic coverage, its immunological impact under field conditions has not been fully characterized. Therefore, the contribution of the vaccination sequence to the observed antibody kinetics could not be determined in the present study. The fluctuations in HI titres are more likely related to multiple factors, including antigenic mismatch with the circulating strain and field-related variability.

Although NA primarily functions during the final stage of infection by facilitating virion release through cleavage of sialic acid receptors, several studies have also implicated NA in viral entry and in shaping immune responses. Therefore, NA should be considered alongside HA in the context of vaccine design [84,85,86,87]. In this context, the AHS-M strain lacked the NA stalk deletions frequently reported among Goose/Guangdong H5N1 lineage viruses. Only three conservative substitutions (Lys217Arg, Ile304Val, Ile394Val) were identified. Among these, Ile304Val has been associated with altered neuraminidase activity and viral fitness [88,89,90,91,92], whereas Lys217Arg was rare, detected in only one additional Egyptian strain. The absence of stalk deletions may indicate a distinct genetic background, although its functional implications remain to be elucidated. Overall, vaccine breakthrough in endemic settings is likely multifactorial, involving antigenic drift, host immune responses, and field-level factors. These findings highlight the need for continued vaccine updates, strengthened surveillance, and timely detection to limit viral persistence and spread.

Several limitations should be acknowledged. First, the passive surveillance design did not include systematic assessment of post-vaccination antibody titres or cross-reactivity between vaccine-induced antibodies and the field isolate, which limits conclusions regarding vaccine effectiveness. In addition, serological data were limited in scope. Although HI titres were measured, these were not available across multiple pre- and post-infection time points, nor against a comprehensive panel of vaccine antigens. The observed increase in HI titres during peak mortality suggests infection-induced boosting; however, the lack of longitudinal sampling restricts accurate evaluation of baseline protective immunity. Furthermore, HI assays were performed using a limited set of vaccine antigens and did not include the homologous AHS-M antigen or the GWT vaccine strain used in the final booster immunisation. Given the antigen-specific nature of HI assays, the absence of these data constrains precise assessment of cross-reactivity and antigenic matching between vaccine-induced antibodies and the circulating virus. Consequently, direct comparison between responses to vaccine strains and the field isolate was not possible, and the extent of antigenic mismatch remains uncertain. In addition, no controlled challenge protection experiments were conducted. As this study is based on field observations, direct evaluation of vaccine-induced protection against the circulating strain could not be performed. While genetic divergence between circulating and vaccine strains was identified, its impact on protective efficacy could not be confirmed in vivo.

Finally, although sequence analysis suggests possible introduction via migratory birds, epidemiological links between wild reservoirs and affected poultry flocks were inferred primarily from genetic data rather than comprehensive field investigations. Taken together, these limitations indicate that the findings should be interpreted with caution. While the results suggest reduced vaccine effectiveness under field conditions, they do not provide definitive evidence of immune escape. Future studies incorporating homologous serological assays and controlled challenge experiments are required to more directly evaluate vaccine efficacy against circulating strains.

## 5. Conclusions

We report a potential breakthrough HPAIV H5N1 clade 2.3.4.4b infection in a fully vaccinated layer flock, potentially consistent with reduced vaccine effectiveness, under field conditions, potentially associated with low HI responses (GM ~3.6 log_2_ against Re14 vaccine strain) and genetic divergence between the circulating strain and vaccine antigens. Inconsistent antibody responses and amino acid substitutions in key HA antigenic regions likely contributed to reduced vaccine protection. Given Egypt’s susceptibility to H5N1 introductions potentially associated with via migratory flyways and its role as a regional hotspot, continuous molecular surveillance, improved vaccine matching, and reinforced biosecurity and quarantine measures are essential to mitigate HPAIV spread and impact.

## Data Availability

The original contributions presented in this study are included in the article. Further inquiries can be directed to the corresponding authors.
